# Open Science at the generative AI turn: An exploratory analysis of challenges and opportunities

**DOI:** 10.1162/qss_a_00337

**Published:** 2025-01-27

**Authors:** Mohammad Hosseini, Serge P. J. M. Horbach, Kristi Holmes, Tony Ross-Hellauer

**Affiliations:** 1 Department of Preventive Medicine, Feinberg School of Medicine, Northwestern University, Chicago, IL, USA; 2 Institute for Science in Society, Radboud University, Nijmegen, The Netherlands; 3 Galter Health Sciences Library and Learning Center, Feinberg School of Medicine, Northwestern University, Chicago, IL, USA; 4 Open and Reproducible Research Group, Know-Center GmbH and Institute for Interactive Systems and Data Science, Graz University of Technology, Graz, Austria

**Keywords:** artificial intelligence, data, impacts, open science, software, workflows

## Abstract

Technology influences Open Science (OS) practices, because conducting science in transparent, accessible, and participatory ways requires tools and platforms for collaboration and sharing results. Due to this relationship, the characteristics of the employed technologies directly impact OS objectives. Generative Artificial Intelligence (GenAI) is increasingly used by researchers for tasks such as text refining, code generation/editing, reviewing literature, and data curation/analysis. Nevertheless, concerns about openness, transparency, and bias suggest that GenAI may benefit from greater engagement with OS. GenAI promises substantial efficiency gains but is currently fraught with limitations that could negatively impact core OS values, such as fairness, transparency, and integrity, and may harm various social actors. In this paper, we explore the possible positive and negative impacts of GenAI on OS. We use the taxonomy within the UNESCO Recommendation on Open Science to systematically explore the intersection of GenAI and OS. We conclude that using GenAI could advance key OS objectives by broadening meaningful access to knowledge, enabling efficient use of infrastructure, improving engagement of societal actors, and enhancing dialogue among knowledge systems. However, due to GenAI’s limitations, it could also compromise the integrity, equity, reproducibility, and reliability of research. Hence, sufficient checks, validation, and critical assessments are essential when incorporating GenAI into research workflows.

## 1. INTRODUCTION

Generative Artificial Intelligence (GenAI) may be defined as “deep-learning models that can generate high-quality text, images, and other content based on the data they were trained on” (Martineau, 2023). Large Language Models (LLMs) are the foundational technology behind GenAI models, including OpenAI’s ChatGPT and Google’s Gemini. LLMs are trained on huge amounts of text and, when used with sophisticated statistical algorithms embedded in GenAI, allow applications to predict and generate responses to input prompts. Combined with generative adversarial networks (GAN) that also take other forms of input, including images, sound, and other unstructured data, and allow processing to output nontextual forms, this creates important new opportunities for research purposes. A lot has been written about the rise of GenAI, including their use in academic contexts (Borowiec, Dikow et al., 2022; Eisenstein, 2023; Krenn, Pollice et al., 2022; Nordling, 2023; Resnik & Hosseini, 2024; Service, 2020). Arguments in favor of using GenAI mostly highlight efficiency gains, while critics are concerned with issues such as systemic errors and biases (Mittermaier, Raza, & Kvedar, 2023); the lack of moral and legal agency and the resulting diffusion of responsibilities and accountabilities (Brożek & Janik, 2019); and the black box problem alluding to the unclarity of the involved process in generating content (Savage, 2022).

This paper aims to contribute to this rapidly growing literature by reflecting on the potential impact of GenAI on another major topic, namely, Open Science (OS). Recently, a short opinion piece was published, claiming that not only does GenAI pose ethical challenges to OS, but OS also increases GenAI’s ability to cause harm (Acion, Rajngewerc et al., 2023). In this paper, we go deeper and adopt a more nuanced approach to expand the debate about potential positive and negative impacts of GenAI on OS. We will use the UNESCO Recommendation on Open Science (UNESCO, 2021) as a guiding document to systematically explore the intersection of GenAI and OS. Our working definition of OS is the one provided by UNESCO’s Recommendation (p. 7):

… open science is defined as an inclusive construct that combines various movements and practices aiming to make multilingual scientific knowledge openly available, accessible and reusable for everyone, to increase scientific collaborations and sharing of information for the benefits of science and society, and to open the processes of scientific knowledge creation, evaluation and communication to societal actors beyond the traditional scientific community. It comprises all scientific disciplines and aspects of scholarly practices, including basic and applied sciences, natural and social sciences and the humanities, and it builds on the following key pillars: open scientific knowledge, open science infrastructures, science communication, open engagement of societal actors and open dialogue with other knowledge systems.

### 1.1. Why Does the Impact of GenAI on OS Matter?

OS has developed into an umbrella term capturing many facets of scholarly work. Openness is considered as a major pillar of science, which serves various functions in research (Hosseini, Senabre Hidalgo et al., 2024b). Indeed, OS has become a position, as a priority for research actors, spurred by new digital possibilities for accessibility, transparency, and participation in research processes, and a growing awareness of issues concerning research integrity (Haven, Gopalakrishna et al., 2022). Among others, OS enables reproducibility and verifiability, facilitates progress in science by allowing others to build on the applied methods and results of research, and benefits the public by sharing information that can impact policy (Resnik, 2023). OS is characterized by UNESCO as being underpinned by values of “quality and integrity” in ensuring scrutiny and making evaluation transparent, “collective benefit” in recognizing research to be a universal public good, “equity and fairness” in promoting fair and equal access to knowledge for all, and “diversity and inclusiveness” in enhancing diversity of knowledge production (UNESCO, 2021, p. 17).

GenAI, meanwhile, has proven more than hype, emerging as a general-purpose technology with substantial economic, social, and policy implications across various domains, including research (Eloundou, Manning et al., 2023). A survey of postdoctoral researchers conducted by *Nature* in the summer of 2023 (*n* = 3,838) showed that 31% of respondents use GenAI chatbots such as ChatGPT in their work (Nordling, 2023). Of these, 63% used chatbots for text refining; 56% for code generation, editing, and troubleshooting; and 29% for finding or summarizing the literature, followed by use cases such as preparing manuscripts (14%), preparing presentation materials (12%), improving experimental protocols (8%), and other uses (7%). Another study has highlighted the potential of GenAI in scientific research, “particularly in administrative, creative, and analytical tasks” (Fecher, Hebing et al., 2023, p. 2). Any technology used by researchers around the globe and expected to impact various facets of the knowledge generation and consumption process will likely also impact (positively or negatively) OS practices.

Various experiments are under way in respect of the use of GenAI in OS workflows (Olavsrud, 2024; Pewter, 2023). Rapid development of GenAI, dovetailed with the identification of their emergent applications in specific contexts (Kojima, Gu et al., 2022), will likely increase experimentation. Such work should be guided by broad considerations of potential benefits and challenges. GenAI is shown to be prone to hallucination, bias, and variability in outputs, so much so that the notion of a “jagged technological frontier” is used to stress that their performance on tasks of similar perceived difficulty can vary widely (Dell’Acqua, McFowland et al., 2023). Improving GenAI and its applications, as well as ensuring their effectiveness for OS, will be ongoing tasks. As we will demonstrate in this paper, even if experimentation with GenAI has the potential to increase the openness, robustness, and integrity of research, it also creates the risk of compromising exactly these core values. Hence, we will map the potential impacts of GenAI on OS, highlighting possible opportunities and challenges.

## 2. METHODS

**Figure 1.**
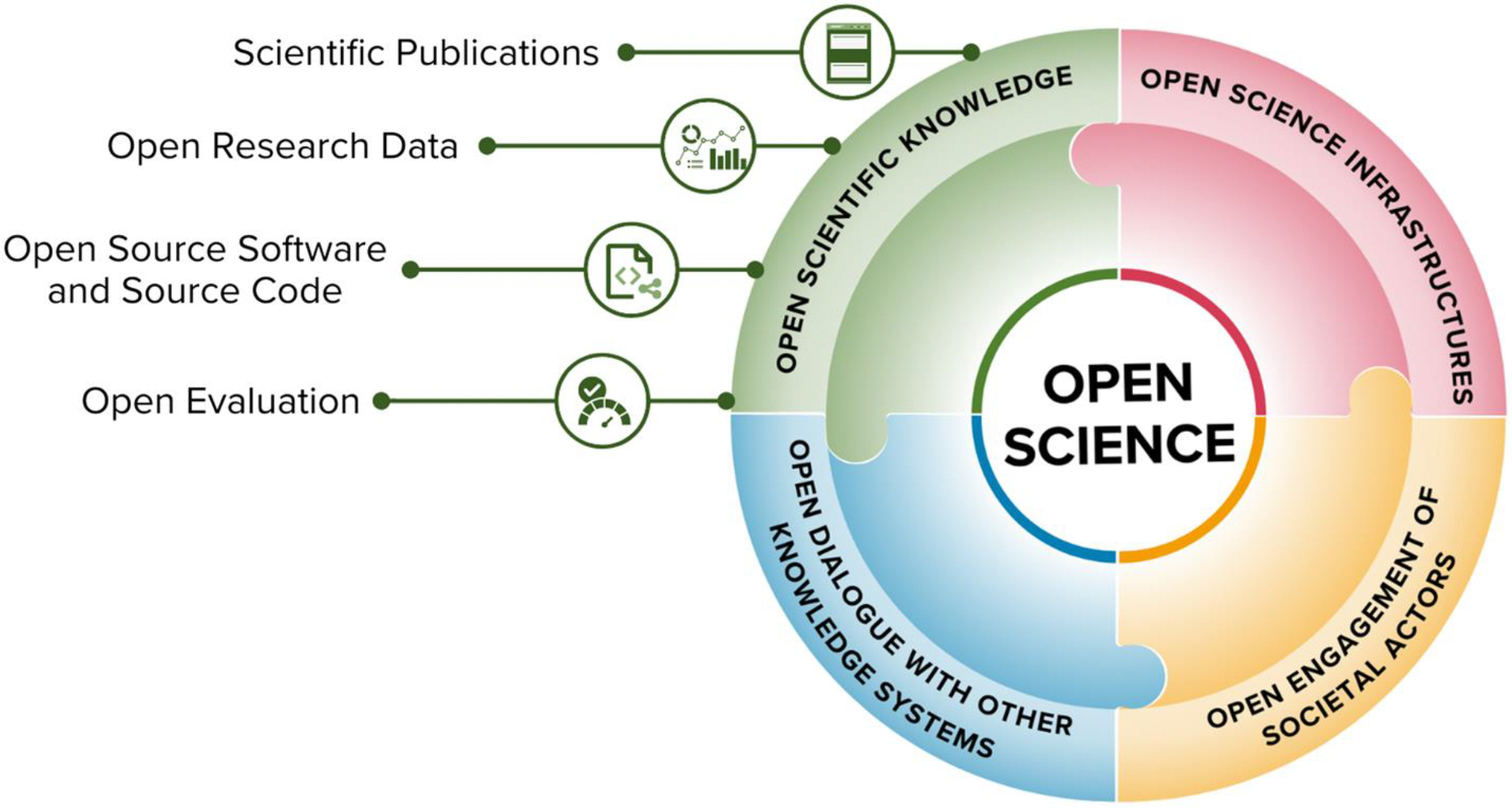
Taxonomy of OS topics, based upon the UNESCO Recommendation on Open Science

In this paper, we limit our discussions to the research-related dimensions of OS, hence omitting elements related, for example, to teaching and education. We used the Taxonomy of OS topics provided in the UNESCO Recommendation on Open Science to structure our discussion of involved issues (UNESCO, 2021). This taxonomy entails four major topical categories, including Open scientific knowledge, Open science infrastructures, Open engagement of societal actors, and Open dialogue with other knowledge systems (Figure 1). We collaboratively discuss the themes within each topic to prioritize those with the highest potential to be impacted by GenAI based on available knowledge and examples in the literature, including those discussed in Resnik and Hosseini’s (2024) work about the impact of AI on norms of science. This led us to a set of seven topics, including scientific publications (Open access), Open research data, Open source software and source code, Open evaluation, Open science infrastructures, Open engagement of societal actors, and Open dialogue with other knowledge systems^1^. In what follows, the impact of GenAI on each theme is explored, followed by a discussion of the broader implications and final conclusions.

## 3. OPEN SCIENTIFIC KNOWLEDGE

Open Scientific Knowledge refers to open access to scientific publications, research data, metadata, open educational resources, software, and source code and hardware that are available in the public domain or under copyright and licensed under an open license that allows access, re-use, repurpose, adaptation and distribution under specific conditions, provided to all actors immediately or as quickly as possible regardless of location, nationality, race, age, gender, income, socio-economic circumstances, career stage, discipline, language, religion, disability, ethnicity or migratory status or any other grounds, and free of charge. It also refers to the possibility of opening research methodologies and evaluation processes. (UNESCO, 2021, p. 9)

### 3.1. Scientific Publications (Open Access)

#### 3.1.1. Possible positive impacts of GenAI

The Open Access movement, in development since the 1990s and crystallized by the Budapest Open Access Initiative (2002), has traditionally focused on making scholarly content freely available for reading and reuse (Tennant, Agrawal et al., 2020). Since then, access to digital content has greatly increased (Digital Science, Hahnel et al., 2023; Morrison, Borges et al., 2022). However, this progress on enabling *physical access* to content arguably spotlights other aspects of what might be considered “open access,” that is, enabling *meaningful* and *equitable* access to that content, both tightly connected to information and data literacy. The former aims to improve conceptual access beyond technical and material access to enable meaningful engagement with open content (Fleerackers, 2023). The latter focuses on ensuring that diverse users, including those without a college degree and with different abilities and cultural and linguistic backgrounds can benefit from open content (Hayes, 1992: Shanahan & Bezuidenhout, 2022; Wentz, Lazar et al., 2021). Enhancing laypeople’s equitable access to published research results is among the expected positive impacts of GenAI (Schmitz, 2023) and aligns neatly with OS objectives.

GenAI can simplify complex scientific concepts, remove jargon, and summarize results, thereby making research publications more accessible to members of the public or researchers from other disciplines. These features could be tailored for multiple nonacademic audiences and purposes. For example, they could support creation of plain language protocols and informed consent documents to better support research subjects’ informed participation in research (Novak, 2024; Xiao, Li et al., 2023), or help digest available research results for policymakers (who may not have the resources or expertise to read scholarly articles and require intermediaries to do the digestion for them), and thus reduce the costs of using science in policymaking (Moon, 2023). While making research digestible for larger groups has been historically the task of science communication experts, GenAI offers a unique feature beyond any science communication expert. Namely, when using GenAI, one can ask questions and demand further clarification *on the spot* if a certain concept or sentence does not make sense or if one needs an example to fully grasp what is meant. This feature enhances cognitive accessibility through offering real-time explanation and support based on specific users’ needs. This could be particularly useful (and perhaps equally dangerous given misinformation campaigns—more on this later), for example, when a member of the public needs urgent medical information for which various contradictory results exist in the literature (Schonfeld, 2023).

These benefits apply not only to nonacademic actors but also to researchers. With an increased rate of knowledge production, recent open access policies such as as PlanS (https://www.coalition-s.org/); the establishment of open infrastructure such as Open Alex (https://openalex.org/); and initiatives to increase the availability of open research information such as the Barcelona Declaration on Open Research Information (2024), have dramatically increased researchers’ access to scholarly information. Furthermore, because openly available information is plentiful and dispersed across various sources and formats, finding useful nuggets of information and using them is getting increasingly more complicated (Hosseini & Holmes, 2023). GenAI can help us address the negative side-effects of this information overload, and support researchers to fully reap the benefits of material access. For example, GenAI trained on specialized knowledge bases can be incorporated in the screening and extraction phases of systematic literature reviews, reducing the burden on researchers and allowing them to widen the scope of their reviews (Bolanos, Salatino et al., 2024). There have also been experiments to finetune GenAI with the previous writings of a specific author to generate text that better resembles the author’s style and tone (Porsdam Mann, Earp et al., 2023). The points about plain language summaries also apply here. GenAI allows researchers to get meaningful access to summaries of content from other fields of which they lack the required expertise to read content directly from research articles.

#### 3.1.2. Possible negative impacts of GenAI

Among the risks of using GenAI to enhance meaningful access to scholarly literature, a significant danger is the potential for systems to provide false or biased syntheses, summaries or advice. GenAI at present is prone to hallucination, errors, randomness, and bias in many areas, including the summarization of literature. Systems poorly deployed, without sufficient testing and safeguards, could hence be disastrous if incorrect information is offered—especially in sensitive or high-risk areas, such as medicine and health. Indeed, while improving the public’s and researchers’ understanding of various scholarly debates is laudable, given the complexity of most scientific topics and the foreseeable inability of nonexperts to fully grasp nuances and limitations (e.g., in the case of experimental health research), GenAI could put the public at great risk (Hoeyer, Couturier et al., 2024). This is more problematic in scientific discourse and topics where a large spectrum of views exists about a certain issue. In these cases, GenAI might be asked to summarize and provide a black-and-white view of the available knowledge, or synthesize a scholarly debate in one paragraph without careful consideration of limitations, all to the public’s detriment. When parties with commercial or other nonacademic interests are equipped with GenAI tools that can search and/or summarize open scholarly content, they could tailor their dissemination based on favorable results or views, such as with the ambition to increase sales or steer public opinion. While this has been a possibility even before GenAI, and is not limited to openly available scholarly content^2^, GenAI’s ability to scale up these efforts combined with increased incentives to make science more open, present an undeniable risk factor. In situations wherein science is highly politicized (e.g., the COVID-19 vaccine), the ability of GenAI to generate personalized content at scale could enable certain actors to alter society’s views—and ultimately decisions—in ways that might not be in the best interest of society. Given that readers cannot always readily assess the quality of presented information, GenAI’s ability to generate content and summaries of research at scale, combined with their ability to use pseudoscientific language for persuasion, could be a recipe for erosion of trust in science. Although some have argued that GenAI could also present solutions in terms of detecting misinformation (Lucas, Uchendu et al., 2023) and deep fakes (Jingnan, 2024), those with expertise in authentication technologies stress that forensics can only do so much (Chennamma & Madhushree, 2023; Gu, Wang et al., 2022).

A second risk of GenAI usage for the production of open content relates to its potential to enable more paper mills (i.e., for-profit entities that fabricate and sell scholarly manuscripts). Before the release of GenAI applications and the wide accessibility of LLMs and the transformer technology, major investments and resources were required to operate a paper mill. Even so, because of their mistakes and nonsensical phrases (AKA “tortured phrases”), academic sleuths were able to spot paper mill productions (Cabanac & Labbé, 2021). However, given GenAI’s accessibility and the release of plugins that can be installed on top of models such as ChatGPT (GPTs) and Claude (Projects), it is much easier (and cheaper) to digest one’s own or others’ content/data and generate seemingly original papers that are potentially more difficult to detect. To the extent that GenAI enables more paper mills and makes detecting paper mill productions more difficult, it increases the signal-to-noise ratio in the scholarly corpus, thereby reducing the findability of high-quality content.

When GenAI is used by researchers to explain and visualize their work, imprecise and unrealistic images can be generated (Kim, Um et al., 2024). For example, a 2024 paper published in the journal of *Frontiers in Cell Development and Biology* depicted a rat with unreal features (Pearson, 2024). Although one might argue that this example simply involved extremely sloppy research (resulting in the quick retraction of the paper), it showed that GenAI’s ability to support researchers may not always be conducive to quality and integrity of research, especially in cases of poor human use (when generating images) and oversight (when the peer review fails in the context of fast turnaround times for commercial benefits). On that note, amplification of the highly competitive “publish or perish” culture could also be a negative side effect of using GenAI, as it enables researchers to publish more with less time and effort (Resnik & Hosseini, 2023). In the context of open access publications, the availability of more regurgitated content (e.g., papers that are identical in essence, but differ in framing or presentation) increases superfluous publications and can worsen the noise to signal ratio and negatively affect the visibility and findability of research.

Finally, given the lack of proper attributions of training data, GenAI challenges the notion of originality and may discourage open sharing of data. Regarding originality, for instance, in humanities disciplines where new forms of expression, novel interpretations, or rhetorical structures (instead of new empirical observations) are among the hallmarks of original research, GenAI’s increased use makes it difficult to determine originality of content because it can rephrase existing content without adding new insights or being caught by plagiarism detection software. When sharing data, attributions are not only accepted and promoted, but are a requirement of data reuse, such as when open licenses such as CC-BY are used. However, lack of proper attribution in GenAIs may discourage the creators of original content from open sharing, fearing that their content will be crawled and used to train commercial models without proper attributions, or worse, be misinterpreted and misused. Indeed, GenAI may inadvertently encourage efforts to make data and information less findable and/or less accessible. Recent efforts of some large corporations such as X (2023) and publishers such as Wolters Kluwer (2021) to limit or completely stop web crawlers and data miners from accessing their data shows signs of an adverse reaction to scraping/crawling efforts. The same reaction can be considered as a last resort and adopted by universities and libraries as well as researchers who might try to prevent their data and results from being used without attribution (Wild, 2024).

### 3.2. Open Research Data

#### 3.2.1. Possible positive impacts of GenAI

Open or FAIR (Findable, Accessible, Interoperable, and Reusable; see Wilkinson, Dumontier et al. (2016)) data is a cornerstone of OS, supporting essential principles, such as transparency, reproducibility, and reusability of research. Open data is freely accessible, reusable, and shareable, typically without restrictions. FAIR data, on the other hand, emphasizes enhancing the reusability and longevity of the data, often (especially in the case of sensitive data) without requirements for openness. The advent of GenAI, offers transformative potential for open and FAIR data practices. Crucial to FAIR data is the implementation of robust Research Data Management (RDM) practices from the onset of a research project. RDM workflows include multiple stages, such as planning, collection, processing, storage, preservation, and sharing. GenAI can potentially facilitate and streamline these stages. GenAI could, for instance, assist in creating data management plans, ensuring unique file-naming and versioning of data sets, and suggesting discipline-specific data repositories. Some exploratory work indicates they may support data validation and cleaning (Alexander, Katz et al., 2023). GenAI could also support data curation and help improve discoverability of digital objects (Levenstein & King, 2024). A keystone of effective sharing is the creation of high-quality metadata. Caliskan, Dangwal, and Dandekar (2023) suggest that while GenAI has limitations and needs continuous monitoring and performance evaluation, it could assist bioinformaticians to annotate metadata or identify discrepancies between metadata and publications. Sundaram and Musen (2023) introduced FAIRMetaText, a tool that uses GenAI to analyze and enhance metadata quality, which could improve the metadata generation process to help ensure FAIR data sets. Patiny and Godin (2023) highlighted GenAI’s potential in extracting experimental data regarding molecules from publications to make new data sets in a cost-effective manner.

GenAI, along with other AI systems, may help identify inconsistencies or irregularities in (open) data indicative of error or fraud (Hill, 2024). GenAI also holds promise in data analysis. The Advanced Data Analysis (ADA) feature of ChatGPT’s GPT-4, for example, may assist users in cleaning, reading, describing, and visualizing data, as well as with advanced statistical analysis. Using GenAI for data analysis has already been investigated, for example, in archival analysis (Hosseini, Hong et al., 2024a), hydrology (Irvine, Halloran, & Brunner, 2023), chemistry (Jablonka, Schwaller et al., 2024), pharmacology (Shin & Ramanathan, 2024), and many more contexts.

Finally, “synthetic” data can be useful in scenarios where real data are unavailable, limited, or too sensitive to share, as it allows for the development and testing of machine learning models without compromising data privacy or security. GenAI and different LLMs have been shown to have great potential here (Giuffrè & Shung, 2023; Ive, 2022; Yoo, Park et al., 2021).

#### 3.2.2. Possible negative impacts of GenAI

High-risk irregularities have been observed when GenAI is used for data analysis. For instance, in line with the known issue of hallucination, when using GenAI for data analysis, Perkins and Roe (2024, p. 3) observed generated “quotes or data that did not exist in the original dataset.” As will be discussed Section 3.3, there is also a danger that in empowering novice scientists with powerful data analysis tools, users who may lack the critical knowledge to assess the accuracy of analyses would generate inaccurate results. Openness of data sets with such issues may have knock-on effects on the integrity and reproducibility of research that builds upon such data or replication studies that require authentic and real data.

While synthetic data can be useful, GenAI can generate entirely artificial/fake data sets. As Taloni, Scorcia, and Giannaccare (2023) have observed, synthetic data sets could also support specific hypotheses. Although these fabricated data sets are identifiable now, as technology becomes more sophisticated, synthetic data could be made openly available without being appropriately labeled, or worse, be misused to falsely validate research in academic papers (Resnik & Hosseini, 2024). In the context of paper mills and other ill-intentioned actors, this is particularly concerning because it allows fraudsters to readily share fabricated data associated with a paper, making it even more difficult to distinguish fake papers. Because, at present, checks for data quality are not part of most peer review or OS monitoring workflows, data availability can sometimes be taken as a surface-level indicator of the transparency, authenticity, or even quality and trustworthiness of research studies *per se*. If fabricated data supporting specific hypotheses becomes easier to produce, formal prepublication checks on data quality will become even more essential.

### 3.3. Open Source Software and Source Code

#### 3.3.1. Possible positive impacts of GenAI

Publicly sharing the code that was used in research is another key plank of OS. GenAI is increasingly being integrated into research coding and software engineering tasks, with, for example, 56% of postdoctoral researchers (who used AI) reporting using GenAI for coding tasks (Nordling, 2023). This has several potential implications for OS. Models such as ChatGPT perform well on coding tasks, with specialized tools already available including Github Copilot and OpenAI Codex. Other models include DeepMind’s AlphaCode (https://alphacode.deepmind.com/), Tabnine (https://www.tabnine.com/), and the open source Polycoder (https://github.com/VHellendoorn/Code-LMs). These tools are capable of translating natural language to code, and can be used to detect errors and suggest code and functions in real time. To the extent that GenAI can democratize coding and support research projects to improve their code, it improves open source software and source code.

Furthermore, by “reviewing” code in real time, such tools can help avoid coding errors and potentially improve code quality and maintainability. This is a major benefit for OS, considering that a recent study analyzing R files associated with over 2,000 replication data sets found coding errors to be common, with 74% of files failing initial execution whereas 56% failed after the simple application of automatic code cleaning (Trisovic, Lau et al., 2022). Assistance from GenAI and associated specialized tools may hence decrease such errors, and potentially contribute to the reproducibility and quality of analyses. GenAI can also assist in generating documentation for code, with specific tools such as Snorkell (https://snorkell.ai/) designed for this purpose. This can positively impact OS because as data show, scientific code is often poorly documented (Rai, Belwal, & Gupta, 2022), despite this being essential for future maintenance and reuse, including future understanding of its purposes and decisions taken in its creation. According to Benureau and Rougier (2018), poor documentation of code is often due to scientists’ insufficient training in software engineering, with potential reuse being an afterthought (Gomes, Pottier et al., 2022).

#### 3.3.2. Possible negative impacts of GenAI

Although GenAI may reduce error rates by checking code and suggesting revisions, it could also add inaccuracies by suggesting code that is unnecessary or unsuitable. This could result from a few factors, such as the “nondeterminism” of outputs (that very different code is suggested for the same prompt), especially as models change over time (Ouyang, Zhang et al., 2023). Inaccuracies may also occur if significant time has passed between the collection of training data and model training, and the use of the model. In that case, the model may suggest code that builds upon software libraries or APIs that have since been deprecated, so that code dependencies no longer work, and neither does the code (Zhong & Wang, 2023). As Ouyang et al. (2023) note, such factors can negatively impact “the reliability and reproducibility of empirical software engineering” and are a potential “menace” to the validity of scientific conclusions. In light of this, critical scrutiny of code generated/modified by GenAI is essential, but this leads to a new concern. While the tools discussed in this section can potentially democratize coding, lowering the bar to entry for those new to coding or those learning new programming languages, it also means that they may lack the critical skills for assessing code (Finnie-Ansley, Denny et al., 2022). In other words, because GenAI empowers scientists who lack the fundamental underlying knowledge to assess what the code does, it could lead to future issues for code reproducibility, especially as reviewing code is still not an established part of peer review workflows (Nüst & Eglen, 2021).

### 3.4. Open Evaluation

#### 3.4.1. Possible positive impacts of GenAI

There is a great appetite to reform how research and researchers are evaluated and assessed. Dissatisfaction with existing practices (e.g., perceived overreliance on inadequate metrics, narrow views on what constitutes value in scholarly work) have resulted in calls to reform scholarly evaluations. Initiatives such as DORA (2012), the Declaration on Research Assessment; CoARA (2022), the Coalition for Advancing Research Assessment; and the Barcelona Declaration on Open Research Information (2024) advocate for responsible assessment and OS practices enabling responsible assessment. GenAI can support these initiatives in compelling ways. For example, GenAI could assist in better identifying and interlinking a broader range of outputs (e.g., data and software), and make them identifiable with better metadata, though currently still at the risk of unreliable linkages. Additionally, by leveraging Multimodal GenAI, which can draw outputs from various data types to provide insights (Wadhwani, 2023), the complex problem of reflecting the true breadth of academic contributions and highlighting a more nuanced understanding of knowledge translation could be easier to address. This could furthermore be supported by GenAI’s ability to detect the presence of data and code, and potentially assess the quality of data and/or perform code review. This in turn provides opportunities to highlight the range of products (e.g., software) involved in research. All of these are, however, subject to the caveats and limitations described in Sections 3.2 and 3.3.

In addition to the potential for contributing to the evaluation of various kinds of open materials and data, locally installed and safe/secure GenAI could also play a role in assessing progress reports or narrative CVs and scholarly manuscripts in peer review contexts. In the former case, GenAI could help conduct an initial triage and support evaluators in summarizing reports and identifying key themes. Open and equitable evaluation aims to ensure that key achievements of a researcher or project are understood and recognized, so an initial triage could be a major benefit.

Elsewhere, we have discussed the potential impact of GenAI on the scholarly peer review system (Hosseini & Horbach, 2023). Although GenAI has the potential to increase efficiency and facilitate contributions of a wider range of actors, funders such as the National Institutes of Health (NIH) (National Institutes of Health, 2023) and the Australian Research Council (Australian Research Council, 2023), as well as publishers such as Elsevier (Elsevier, n.d.) have banned its use in peer review to prevent confidentiality issues and avoid biased and erroneous reviews. Therefore, among practices related to peer review, the benefits of GenAI are currently relevant to actors that allow its use.

Apart from these general considerations regarding the impact of GenAI on scholarly peer review, these new technologies also have specific implications for Open Peer Review (OPR), which is among the OS pillars. OPR can signify various practices and models of peer review. Most commonly, OPR refers to the use of open reports (publishing review reports alongside manuscripts), open identities (disclosing reviewers’ identities to authors and readers of manuscripts), and open participation (involving noninvited reviewers, potentially also nonacademic stakeholders) (Ross-Hellauer & Horbach, 2024). From the discussions in previous sections, it is clear that GenAI has the potential to strengthen open participation by facilitating contributions from a wider range of actors, including those usually excluded from the review process, such as patients, research participants, and other nonacademic actors affected by the research. While several publishers and journals are currently banning the use of GenAI tools for review, other peer review contexts are not as strictly regulated (e.g., postpublication peer review involving preprint servers and/or peer review platforms). In these contexts, which sometimes have the explicit aim to diversify the reviewer pool, GenAI can facilitate the contribution of new actors to the process. Hence, in terms of OPR, the most significant positive contribution of GenAI should be expected in terms of more inclusive and equitable participation.

#### 3.4.2. Possible negative impacts of GenAI

In contrast, GenAI’s potential impact on open review reports can be concerning. OPR reports have been suggested as an effective remedy against predatory journals (Yamada, 2021) and have enabled metaresearch on peer review (Buljan, Garcia-Costa et al., 2020; Sizo, Lino et al., 2019). As GenAI can instantaneously produce superficial (or even meaningless) but seemingly convincing review reports, when used by malicious actors to generate review reports it could increase the legitimacy of predatory journals. Hence, the mere publication of OPR reports might no longer suffice to demonstrate that meaningful peer review has taken place. Additional measures are needed to assure readers that manuscripts have actually been reviewed—for example, a system of open identities, indicating who has written the review and when. There are, however, legitimate concerns about the understudied negative implications that such a system of open identities might have, for example in terms of retaliation following critical reviews (Ross-Hellauer & Horbach, 2024). This could particularly affect reviewers in vulnerable positions (e.g., early-career scholars or members of minority groups), thereby potentially decreasing the diversity of the reviewer pool and undoing the positive contributions outlined in the previous subsection.

In addition, as mentioned before, several funders and publishers have cast doubt on the use of GenAI for review and assessment purposes due to the risk of breaching confidentiality. This could be especially relevant for work that is not openly available or or is in progress, including grant proposals. While some of these concerns might be addressed by using local instances of GenAI (that neither feed data into training LLMs nor allow access to outside users), this is a costly and resource-intensive solution (Arancibia, 2024), which can probably only be justified in cases where evaluation happens at scale and does not require external sources of information. Notably, this is frequently *not* an equitably available solution for individual evaluators or organizations. Either way, analyzing research output and progress reports with GenAI (be it in the cloud or local) requires permission from institutions and individual researchers, along with a specific process involving humans to ensure accuracy and accountability for the evaluation. Currently, there are no infrastructures or guidelines for this purpose.

## 4. OPEN SCIENCE INFRASTRUCTURES

Open science infrastructures refer to shared research infrastructures (virtual or physical, including major scientific equipment or sets of instruments, knowledge-based resources such as collections, journals and open access publication platforms, repositories, archives and scientific data, current research information systems, open bibliometrics and scientometrics systems for assessing and analyzing scientific domains, open computational and data manipulation service infrastructures that enable collaborative and multidisciplinary data analysis and digital infrastructures) that are needed to support open science and serve the needs of different communities. … Open science infrastructures are often the result of community-building efforts, which are crucial for their longterm sustainability and therefore should be not-for-profit and guarantee permanent and unrestricted access to all public to the largest extent possible. (UNESCO, 2021)

### 4.1. Possible Positive Impacts of GenAI

GenAI, via many mentioned use cases here, presents new opportunities for research infrastructures to either streamline and share existing workflows or create new ones. Numerous examples of innovation powered through open infrastructure exist (OpenInfra Foundation, n.d.), offering the research and scholarly community the opportunity to consider their work and processes in new ways and readily share available infrastructure. More broadly, OS practices such as research data management, access to a wide range of digital research objects, and reinforcement of FAIR practices, depend on widely available OS infrastructures (Schonfeld, 2019). Generalist repositories, such as Zenodo and Dataverse, accept deposits of data and other digital research objects in varying sizes, domains, and file types. Coordination by infrastructure resources such as generalist repositories creates new opportunities for collaboration (GREI Zenodo Community, n.d.; GREI Community, 2024), reinforcement of FAIR Practices, and alignment of “a common set of cohesive and consistent capabilities, services, metrics, and social infrastructure” required by researchers (NIH Office of Data Science Strategy, 2024). GenAI can play a significant role in efficient use of open repositories, by streamlining curation and documentation workflows (NIH Office of Data Science Strategy, 2023).

Furthermore, by automating containerization (i.e., bundling an application and its associated files into a single package to run on different infrastructures), GenAI can streamline the sharing and deployment of tools across platforms, enhancing data sharing and replication as well as the portability and scalability of computational resources (opea-project/GenAIInfra, 2024).

Finally, the openness of research information, upon which to base assessments, is of increasing significance, as demonstrated by efforts such as the Barcelona Declaration on Open Research Information. Central to the effective sharing of research and its impact are infrastructures such as Scopus, Google Scholar and Web of Science (WoS), which serve as foundational elements in the scholarly ecosystem. Yet, there is a burgeoning movement advocating for the *reclaiming* of openness in these infrastructures. Movements for Open Citations and Open Abstracts have gathered pace (van Eck & Waltman, 2022), and in recent years attempts to create an open alternative to Scopus, WoS, or Google Scholar have resulted in OpenAlex (https://openalex.org/). Supporting this openness will be a further boon to GenAI models, as the incorporation of good quality structured metadata and metadata enrichment efforts (e.g., Buttrick, 2024) will add further robustness as either model training data or as augmented information in neuro-symbolic models^3^, although notably, the possibility of monetizing closed databases as training data for AI applications could further restrict reuse rights in those cases.

### 4.2. Possible Negative Impacts of GenAI

A key issue in GenAI, *qua potential* infrastructure for OS, is the fact that many of the most prominent current models are themselves not “open.” Even among projects claiming to be open source, “many inherit undocumented data of dubious legality,” “few share the all-important instruction tuning (a key site where human annotation labour is involved),” and “careful scientific documentation is exceedingly rare” (Liesenfeld, Lopez, & Dingemanse, 2023, p. 1). Liesenfeld and colleagues maintain a “Live Tracker” of LLMs’ openness (https://opening-up-chatgpt.github.io/), which currently shows the most popular LLM (i.e., OpenAI’s GPT), residing firmly at the bottom of the list with a complete lack of availability of training data; open code; LLM weights or reinforcement learning data; model cards; or data sheets. Given this lack of transparency, the extent to which profoundly nonopen GenAI tools, such as OpenAI’s ChatGPT, are fit for purpose as a tool for open research remains questionable (we return to this theme in Section 7.3).

Additionally, copyrights, licensing, and, more broadly, legal issues are among the challenges of using GenAI that remain unresolved. Developers of LLMs such as GPT-4 have not yet disclosed the sources they used to train these models. As a result, generated content lacks proper attribution and users always run the risk of infringing copyrights or committing plagiarism. Obscurities in terms of provenance and sources of information and the risks associated with illegal and fraudulent activities challenge the interoperability of data and knowledge (Douthit, Fiol et al., 2021; Geisler, Vidal et al., 2021). Accordingly, skepticism and hesitation to use generated content may challenge one of the core objectives of Open Science, namely scientific progress. Furthermore, given that LLMs have used open content as training material, they affect publishers’ business models (e.g., generated summaries and paraphrases minimize the need for users’ direct access, thereby limiting publishers’ ability to collect user engagement data on their platforms) as well as the viability of volunteer-based websites such as Wikipedia (e.g., through decreased traffic and engagement, and lack of proper attribution).

## 5. OPEN ENGAGEMENT OF SOCIETAL ACTORS

Open engagement of societal actors refers to extended collaboration between scientists and societal actors beyond the scientific community, by opening up practices and tools that are part of the research cycle and by making the scientific process more inclusive and accessible to the broader inquiring society based on new forms of collaboration and work such as crowdfunding, crowdsourcing and scientific volunteering. (UNESCO, 2021)

### 5.1. Possible Positive Impacts of GenAI

As noted in Section 3.1, GenAI can facilitate *meaningful* access to scientific content, particularly through plain-language summaries or other derivative text, which is arguably one of the main prerequisites of meaningful engagement of societal actors. This enhanced access to academic content can subsequently create opportunities for societal actors, be they in professional roles (such as policymakers) or in individuals’ private capacity, to engage in scholarly debates, for example by understanding, engaging with, or commenting on scholarly articles. This increased opportunity for dialogue can take multiple forms, including citizens asking questions or seeking clarifications, helping to set agendas, and establishing research priorities. More generally, the use of GenAI by societal actors can enhance communication, facilitating clearer two-way interaction between researchers and the wider public as well as inviting nonexpert participation in scientific discussions, bringing diverse insights and potentially fostering a more inclusive research environment. As noted, GenAI can facilitate such engagement by enabling basic understandings of complex topics to nonexperts as well as by removing or lowering language and jargon barriers. This can contribute to the long asked-for calls to the research community to increase citizen and societal participation in research, by moving from merely informing, to more genuine partnerships (Losi, 2023). In short, taking Arnstein’s ladder of citizen participation levels ranging from manipulation to citizen control (Arnstein, 1969), GenAI could facilitate the move to higher levels of citizen engagement in research processes.

Furthermore, GenAI could enhance citizen science initiatives by empowering participants to engage in more complex and meaningful tasks, thereby addressing common critiques such as the “data drone” concern, where citizens are seen as mere data collectors rather than active researchers (Strasser, Baudry et al., 2019). GenAI can serve as an intermediary, translating complex scientific concepts into understandable language as well as automating part of the research process, thus enabling citizens to contribute more substantively to data analysis and interpretation. Especially in contexts such as nature conservation, where it is necessary to guide citizens through sophisticated protocols, answer questions, and provide explanations to make the research process more accessible and enrich educational experiences (Ganzevoort & van den Born, 2020), GenAI can be helpful. This can also potentially contribute to more fully engaging citizens beyond the range of “usual suspects” that tend to contribute most to citizen science projects (Ganzevoort, van den Born et al., 2017). Moreover, by incorporating advanced error-checking and data validation algorithms, GenAI can improve the accuracy and reliability of the contributions, mitigating concerns about the quality of citizen-collected data (Finger, van den Bogaert et al., 2023). When implemented carefully, this can elevate the role of citizen scientists from passive data collectors to active and informed participants and contributors, and can create opportunities for collaboration, reinforce collaborations, and enhance the overall quality and credibility of the research outcomes.

### 5.2. Possible Negative Impacts of GenAI

Among the potential negative impacts of GenAI on engagement of societal actors are several issues we have covered earlier in this work, including the risk of spreading misinformation; an overreliance on technology that may lead to a decline in critical thinking and reduced direct human engagement; bias and representation issues, potentially skewing the public understanding and perception of research; and accessibility issues related to the digital divide, risking exacerbating existing inequalities if access to GenAI is limited to certain groups or regions.

## 6. OPEN DIALOGUE WITH OTHER KNOWLEDGE SYSTEMS

Open dialogue with other knowledge systems refers to the dialogue between different knowledge holders, that recognizes the richness of diverse knowledge systems and epistemologies and diversity of knowledge producers [… and] aims to promote the inclusion of knowledge from traditionally marginalized scholars and enhance inter-relationships and complementarities between diverse epistemologies, adherence to international human rights norms and standards, respect for knowledge sovereignty and governance, and the recognition of rights of knowledge holders to receive a fair and equitable share of benefits that may arise from the utilization of their knowledge. (UNESCO, 2021)

### 6.1. Possible Positive Impacts of GenAI

In distinction to many other definitions, the UNESCO definition of OS places increased emphasis on dialogue with other knowledge systems, to recognize “the richness of diverse knowledge systems.” We anticipate a potential role for GenAI in acting as a switchboard between diverse knowledge systems (e.g., between indigenous and formal science or in the context of medicine, between traditional/herbal/holistic medicine and modern medicine), thereby facilitating dialogue and knowledge transfer (Richards, Worden et al., 2024). This can be achieved through the simplification of complex concepts, removal of jargon, and suggesting context-specific examples to facilitate dialogue between different knowledge systems.

In addition, supporting and enhancing translation is a potential value of GenAI for opening a dialogue between various scholarly communities. While some might argue, based for example on standard journal metrics, that the majority of reputable scientific journals are published in English, not everyone has a good command of this language. Translation of research results published in various foreign languages could also open up scholarly fields in different countries and enhance possibilities for international collaborations. This could include enhanced collaboration across parties of interest (e.g., policymakers and community members) and more inclusive forms of research communication, such as plain language summaries (Dormer, Schindler et al., 2022). Especially for laypeople, reading content in another language might be difficult, if not impossible. Although free online translation services such as Google Translate have been available for a while, GenAI can support and enhance cognitive accessibility and *dialogue* beyond a translation website, because they accommodate real-time interaction, allowing users to ask context-specific questions about the translated text. As, in many contexts, research is mostly funded by taxpayers’ money, GenAI’s enhancement of equitable access to research publications aligns with core Open Science values and researchers’ accountability to the public.

### 6.2. Possible Negative Impacts of GenAI

However, GenAI arguably poses risks to an open dialogue. On the one hand, the opacity of data used to train many GenAI models poses a risk that available knowledge is used in ways at odds with the FAIR (Wilkinson et al., 2016) and CARE principles (Carroll, Garba et al., 2020). Because GenAI reflects the training data and values encoded in the human feedback used for fine tuning or algorithms used for reinforcement learning (Franceschelli & Musolesi, 2024), GenAI will likely reflect hegemonies of who is most visible on the internet, in terms of the regions, languages, and demographics or worldviews of those in charge of its training. GenAI is known to inherit and potentially amplify human biases from training data related to race, gender, ethnicity, and socioeconomic status (Larkin, 2024). A recent study that investigated OpenAI’s Application Programming Interface (API) to generate responses on psychological measures with cross-cultural survey data showed that their “performance on cognitive psychological tasks most resembles that of people from Western, Educated, Industrialized, Rich, and Democratic (WEIRD) societies but declines rapidly as we move away from these populations” (Atari, Xue et al., 2023). Hence using GenAI may stand to further encode epistemic hegemonies and perpetuate/amplify biases in scientific interpretation.

Another negative side effect pertains to translation of scholarly content. While GenAI’s ability to translate content increases access, it is also among their pain points. This is partly because of the intricacies of translation in general (e.g., certain concepts are not possible to translate or require human judgment to identify context and the correct form), and partly because of algorithms’ shortcomings in translating content in a manner comparable to what human translators can do (Dentella, Guenther et al., 2023). More importantly, as different languages have not been equally represented and used when training GenAI, significant challenges in translations are likely to persist for the time being (Ta & Lee, 2023). As a result of poor-quality translation of scholarly content, concepts and even facts could be miscommunicated or misrepresented in languages other than the source language, thereby reducing content accuracy in different languages.

## 7. DISCUSSION AND CONCLUSION

### 7.1. Common Themes

**Figure 2.**
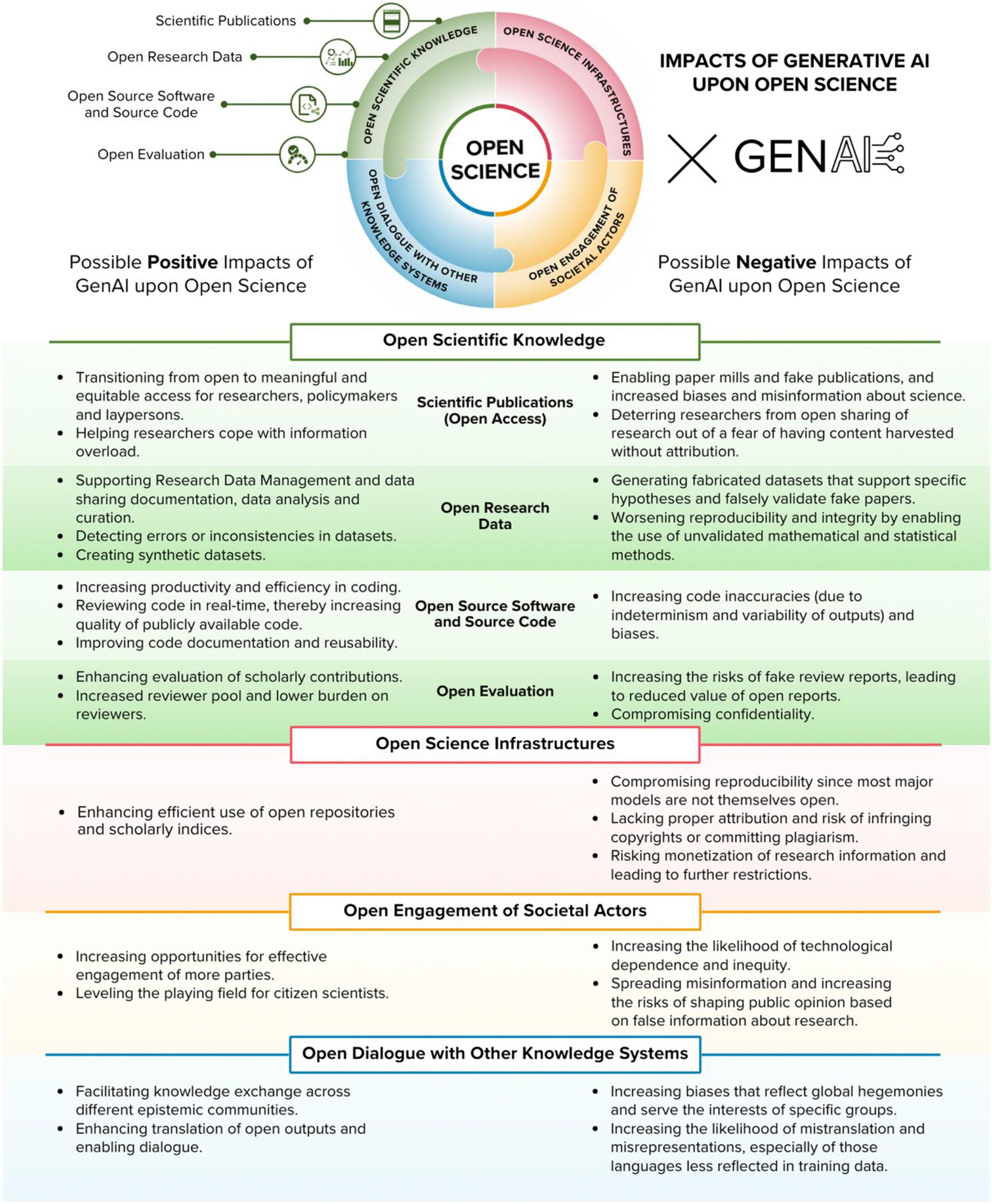
A summary of possible positive and negative impacts of GenAI on OS.

GenAI poses potential positive and negative impacts on OS (see Figure 2 for a summary).

In our discussion of the potential impacts, both positive and negative, of GenAI on OS practices, three key themes emerge. The first relates to what we have called *meaningful access*. The OS movement has long campaigned for increased access to research products and materials, including open access articles and open data. However, these initiatives frequently faced the challenge of research products usually not being written or shared in a format or language that allowed citizens to understand or meaningfully engage with them (Evans & Campos, 2013). While GenAI can facilitate meaningful and equitable access, it is not risk free. Indeed, such usage creates a form of epistemic dependence (Russo, Schliesser, & Wagemans, 2024), where many users might not be able to critically verify GenAI’s outputs, on top of the risk of intentional or strategic misuse of scholarly content. The risk of *creation and spread of misinformation or disinformation*, fueled by GenAI, constitutes the second recurring theme in our discussions. Third, GenAI has *potential implications for diversity, equity, and inclusivity* of academic research, which are specifically significant for OS. An increase in meaningful access to academic output and materials and increased possibilities for meaningful engagement with the research process potentially foster inclusivity and promote a more diverse set of actors to engage with research. However, concerns about biases ingrained in the inner workings and/or training data of GenAI could simultaneously harm OS objectives in terms of propagating certain worldviews or ideologies while dismissing or ignoring others.

### 7.2. Is It Worth Using GenAI in OS Workflows?

GenAI partially fulfills some of the early promises of the Open Data movement—where many, including policymakers, framed data as the “new oil” (Leonelli, 2023, p. 48; Wessels, Finn et al., 2017, p. 161)—through its heavy use of OS outputs including open access literature, open data, and open code, for training data^4^. But the realities, touched upon above, of (often closed) commercial models and resource-intensive developments, in addition to the issues of potential bias and error in results, pose critical questions for the overall move towards incorporation of these tools into OS workflows. It is worth expanding on these issues as we close this paper.

The resource intensity of GenAI, including the accumulation of increasingly large amounts of training data, and the financial and human costs of training, finetuning, and running these models, requires skills and training to use them effectively, and the huge ecological footprint associated with their use has profound implications for equity of access to these tools, and hence, diversity and inclusion in their use (Arancibia, 2024; Maslej, Fattorini et al., 2024; Rowe, 2023). As GenAI tools become more established, the political economy of platforms, where larger developers gain monopoly and extend their advantage, will likely compound these issues. Especially if closed and privately run models become core infrastructure for OS, this may further exacerbate the dynamics of exclusion and cumulative advantage that are already known to be at play within OS (Hosseini et al., 2024b; Ross-Hellauer, Reichmann et al., 2022).

Given the aforementioned issues, and especially the tremendous environmental and economic costs of GenAI (Chien, 2023), researchers must be particularly vigilant in raising the question of whether these tools are the most efficient or necessary way of achieving openness in science, or whether there might be cheaper or more environmentally friendly solutions that can serve the same purposes. Given the current period of rich experimentation regarding the potential uses of GenAI, researchers should continually question whether GenAI is the most sensible solution to the problems at hand, considering costs, limitations, capabilities, and the overall balance of benefits versus resource demands, as well as risks.

Finally, the extent to which GenAI model developers and users should generally prefer open source solutions is currently a key topic. As said, many of the most popular models are currently not open in crucial ways. In late 2023, at a global AI Safety Summit hosted by the UK government, the open source question emerged as a key line of division. While some emphasized the risks of misuse and lack of control in open models (Burki, 2024), others focused on the need for open source as a basis for the transparency, explainability, equity, and inclusivity of models and their future development (Fay, 2023). To align with the key ethos of OS (transparency, accessibility, inclusion), we suggest that research communities must prefer open models wherever possible.

### 7.3. Can OS Open Up GenAI?

There is clearly a complex interplay between the potential of GenAI to advance OS and serious risks and challenges that must be overcome. We hence call for a concerted effort among research communities interested to investigate and address these issues, ensuring that GenAI contributes positively to the scientific community, society at large, and meaningful interactions between the two. Put simply, overreliance on GenAI outputs without verification could compromise the integrity, equity, diversity, reproducibility, and reliability of research. Hence sufficient checks, validation, and critical assessments are essential when incorporating GenAI into research workflows.

We argue for the general need to apply open principles to the governance of GenAI, hence bringing openness (back) to GenAI. The OS movement has over the past decades established the infrastructure and vocabulary that can facilitate the responsible development and implementation of GenAI for research purposes. The research community should especially endorse core OS values of fairness, transparency accessibility, and participatory governance to spread benefits in an equitable way and to ensure responsible governance of tools that may soon come to be seen as crucial infrastructure. Towards this end, using GenAI models that comply with OS values (e.g., those that have transparently disclosed sources used in training) would be helpful in encouraging GenAI developers to engage with OS in a more meaningful way.

### 7.4. Recommendations

Before using GenAI, researchers should

- Sufficiently test these tools and employ appropriate safeguards to identify and mitigate risks of bias, error, or randomness in relevant contexts to protect parties involved and promote trust in science.
- Undertake work with a critical eye to the resource-intensive nature of these systems and confirm that using GenAI is the most effective/sustainable solution for the envisioned use case(s).
- Seek and employ open source GenAI models to align with OS principles, and to best ensure fairness, explainability, transparency, and reproducibility.
- Be aware that they are responsible and accountable for GenAI output, and their use of these models should be responsibly and transparently cited (including, where beneficial, information on model version, date of use, used prompts, and any checks for robustness used) to enable open monitoring of performance and the impacts of these models on knowledge production.

In light of GenAI’s impacts on OS practices, institutions, funders, publishers, and others involved in the knowledge production ecosystem, should

- Provide sufficient training and openly available guidance on the strengths and limitations of specific GenAI models.
- Investigate and monitor the potential positive and negative impacts of GenAI on OS in their own contexts, as described in Figure 2.
- Continually monitor and report usage and outputs of GenAI in their own context, especially when contributing to openly available knowledge, data, or infrastructure.
- Investigate capacities for GenAI to improve identification and interlinking of a broader range of outputs (e.g., data and software) for evaluation purposes, as well as potentially assisting in assessing progress reports, narrative CVs, and manuscripts. This must take account of issues of sensitive data and hence prefer locally installed environments, and give due care to inherent biases, randomness, and error.
- Ensure that any use of GenAI for assistance in evaluation is undertaken with appropriate permissions from those evaluated, respect for data protection, and sufficient “human-in-the-loop” safeguards.
- Monitor and counteract the ways in which GenAI may reinforce epistemic hegemonies and perpetuate/amplify biases in scientific interpretation or translation of knowledge.

Given the potential benefits, and especially the risks of GenAI for future OS workflows, the research community should consider and reflect on

- GenAI’s potential to fuel misinformation masqueraded as openly available knowledge. Addressing this requires special attention to at least three areas: paper mills and predatory publishing (e.g., fake papers, data sets, or peer reviews); contentious scientific debates that have been politicized (e.g., climate change or vaccines); and effects of biases on scientific engagement and understanding.
- GenAI’s potential chilling effects on discussions about readiness to share data (e.g., misuse of open content by GenAI).

## Footnotes

1 Under “Open scientific knowledge,” we have omitted “Open hardware” given the lack of GenAI use cases (beyond the speculation that they may in the future be able to assist in hardware design tasks), and “Open Educational Resources,” as our focus here is on the impacts of GenAI upon the research-related dimensions of OS. Under the Open scientific knowledge category, we have included “Open evaluation,” an element usually present within OS debates currently absent in the UNESCO taxonomy.

2 For example, long before GenAI was introduced, Purdue Pharma used a five-sentence letter published in the *New England Journal of Medicine* in 1980 as evidence that opioids are nonaddictive (Leung, Macdonald et al., 2017).

3 Models combining methods of traditional symbolic AI and Machine Learning. See Sheth, Roy, and Gaur (2023) for an introduction.

4 The Mozilla Foundation has recently reported on the “outsized importance” in training LLMs (Baack, 2024; Mozilla Foundation, 2024) of the nonprofit organization Common Crawl (https://commoncrawl.org/) and its huge (9.5 petabytes) open data set of data crawled from the web. More than 80% of GPT-3’s tokens, for example, came from this source (Brown, Mann et al., 2020). Common Crawl and derivative data sets at that time built heavily upon scholarly Open Access, with content from the publishers PLOS, Frontiers, and Springer, and from PubMed Central all among the top-25 most-used data sources (Dodge, Sap et al., 2021). Note, however, that their relative importance may have diminished more recently (cf., list of Common Crawl’s most-crawled domains: https://commoncrawl.github.io/cc-crawl-statistics/plots/domains).
